# Extraction and Characterization of Phenolic Acid Compounds of Zahidi and Khastawi Dates Seed Extract and Evaluation of their Antibacterial Activity

**DOI:** 10.34172/aim.33929

**Published:** 2025-04-01

**Authors:** Noor Ridha Kadhim, Mohammad Rabbani Khorasgani, Hussam Sami Awayid, Hamid Noorbakhsh

**Affiliations:** ^1^Department of Cell and Molecular Biology & Microbiology, Faculty of Biological Sciences and Technology, University of Isfahan, Isfahan, Iran; ^2^Department of Medical Laboratory, Institute of Technical Suwaira, Middle Technical University, Wasit, Iraq

**Keywords:** Date seed, Drug resistance, Methicillin-resistant, Phenolic acid, Staphylococcus aureus

## Abstract

**Background:**

The date fruit is a remarkable source of nutraceuticals and bioactive compounds. Different types of phenolic compounds with high antioxidant capacity are found in date seed extract. Additionally, these compounds can be potential antibacterial agents to combat antibiotic resistance strains. Therefore, the main idea of the current study was to quantify five key phenolic acids in the ethanolic extract of Zahidi and Khastawi dates seed and to examine their antibacterial activity against methicillin-resistant *Staphylococcus aureus* (MRSA).

**Methods:**

The concentration of gallic acid, ferulic acid, p-coumaric acid, sinapic acid and cinnamic acid in the seed extracts were determined by high performance liquid chromatography (HPLC). Then, antibacterial activity of date seed extracts was analyzed using well diffusion method. Furthermore, a scanning electron microscopy (SEM) analysis was performed to confirm the antibacterial effects of the seed extracts.

**Results:**

Phenolic acids were found to be in the range of 10.59 to 33.65 µg mg^-1^ in Zahidi and 13.69 to 41.56 µg mg^-1^ in Khastawi date seed extract. Gallic acid was the dominant phenolic acid, while cinnamic acid was seen in the lowest concentrations compared with the other phenolic acids in both cultivars. Antibacterial activity study showed that the growth inhibition effect of Khastawi date (14±0.21 mm) was higher than that of Zahidi date (8±0.13 mm) against MRSA. As well, the maximum DPPH scavenging percentage was 79% and 62% for the Khastawi and Zahidi date seed extracts, respectively. Also, SEM analysis suggested that treatment of MRSA with date seed extract resulted in a significant disruption of bacterial structure.

**Conclusion:**

It can be concluded that date seed extract can be considered as a potential source of antibacterial compounds for the drug discovery purposes.

## Introduction

 Date palm (*Phoenix dactylifera) *is a perennial woody plant which belongs to Arecaceace and is cultivated in hot and dry regions. Egypt and Iran are the two major producers of date palm followed by Arabian Peninsula countries. Date palm is considered as a nutritious fruit providing a complete diet with applications in traditional medicine systems in many parts of the world.^1^ Apart from carbohydrates, lipids, dietary fiber (β-glucans, arabinoxylans, and cellulose), vitamins (thiamine, riboflavin, E and C) and minerals (K, Ca and Mg), the fruit is a rich source of biologically active compounds. The functional effects of various biomolecules such as anthocyanins, phenolics, sterols, carotenoids, flavonoids, tocopherols, tocotrienols, tannins, phytosterols, phytoestrogens, and saponins in date palm have been characterized in its health beneficial traits like antioxidant, anti-inflammatory, anticancer, antidiabetic, antimicrobial and antitoxic activities in many publications.^2-5^ Extreme climate conditions in which the date palm trees are cultivated such as very high temperature and strong sunlight exposure lead to increase phenolic constituents in the plant.^5^ Phenolic compounds are identified as the plant secondary metabolites involving in the H_2_O_2_ scavenging within the cells.^6^ However, the phenolic profile in date palms differs in nature and titer depending on the cultivar, growth stage and environmental parameters. The high capacity of antioxidant properties confirmed in phenolic compounds, including flavonoid and non-flavonoid (phenolic acids) groups have made these chemicals remarkable nutraceuticals.^7^ To date, a number of studies have investigated the phenolic compounds including hydroxycinnamic acid derivatives, hydroxybenzoic acid derivatives and flavonoids in different section of date palm including the fruit, seed and pollen.^8-13^ Also, the antimicrobial activity of the phenolic extracts of various date palm cultivars have been confirmed against a number of opportunistic pathogens such as *Staphylococcus aureus*, *Escherichia coli*, *Candida albicans, Bacillus subtilis, Bacillus cereus, Enterococcus faecalis, Micrococcus luteus, Listeria monocytogenes, Salmonella Enteritidis, Salmonella typhimurium, Klebsiella pneumoniae, Aspergillus niger and Fusarium spp.*^7,14,15^ *S. aureus* is a gram-positive, opportunistic bacterial pathogen responsible for a wide spectrum of clinical manifestations in human.^16^

 The date tree is native to the Middle East, and in countries such as Saudi Arabia, the United Arab Emirates, and Egypt, date seeds have long been part of traditional medicine. Research in these countries has primarily focused on the antimicrobial and antioxidant activities of date seed extract. While date palm cultivation is not widespread in Europe, North America, and East Asia, there is a growing interest in the use of date palm seed extract in global research due to increased antimicrobial resistance.^17,18^ Developing resistance mechanisms to methicillin and methicillin-derivatives has resulted in emergence of methicillin resistant *S. aureus* strains which demonstrate relatively high mortality and morbidity frequency.^19^ As mentioned above, the type and quantity of phenolic compounds varies among different date palm cultivars which affects their biological activities. The main idea of the current study was to analyze the content of phenolic compounds in seed extracts of two different date cultivars. Another major goal of this study was to find out whether there is a clear relationship between the phenolic content and the antimicrobial and antioxidant properties of date cultivars.

## Materials and Methods

###  Bacterial Strains

 Fifteen methicillin-resistant *Staphylococcus aureus* (MRSA) strains were used in the current study. These bacterial strains were obtained recently during a project on isolation of MRSA from the hospital infectious samples (data not published). Nutrient agar (Sigma-Aldrich, Germany) medium was prepared according to the manufacturer’s instructions and autoclaved at 121 °C for 15 minutes. The cells were revived on nutrient agar and incubated at 37 °C for 24 hours. The plates were preserved at 4 °C until use.

###  Chemicals 

 All chemicals, reagents, and standards used in this study were of analytical grade. Phenolic acids including ferulic acid, cinnamic acid, sinapic acid, gallic acid and p-coumaric acid were purchased from Sigma-Aldrich and ethanol was obtained from Merck.

###  Plant Materials

 Two different cultivars of *P. dactylifera*, Zahidi and Khastawi were provided from local stores in middle and south regions of Iraq. These two species account for the largest amount of date production in Iraq.

###  Preparation of Date Seed Powder

 The seeds were separated from the fruits and washed carefully with dH_2_O to remove any adhering flesh. Then, the seeds were dried at 50°C in the oven for 24 hours and ground to a fine powder by grinder. Prepared date seed powders were stored in sealed glass containers at 4°C for further experiments.^20^

###  Preparation of Date Seed Extract

 Solvent extraction was done in a Soxhlet extraction apparatus. To prepare the palm date seed extract, 10 g of each seed powder was added to 100 mL of absolute ethanol in the thimble and heated at 40-60 °C for 7 hours. Then, the solution was filtered twice through a Whitman No. 1 filter paper and evaporated under vacuum at 40 °C with a rotary evaporator to remove ethanol. Finally, the viscous residue was collected and kept at 4 °C for further use.^21^

###  HPLC Analysis of Phenolic Profile

 Quantification of individual phenolic acids was performed by reversed phase high performance liquid chromatography (HPLC) analysis, using a Sykam HPLC chromatographic system equipped with a UV detector (Zorbax Eclipse Plus-C18-OSD) (25 cm × 4.6 mm). The column temperature was 30 ºC. The gradient elution method, with eluent A (methanol) and eluent B (1% formic acid in water (v v^-1^)) was performed, as follows: initial 0-4 minutes, 40 % B; 4-10 minutes, 50 % B; and flow-rate of 0.7 mL/min. The injected volume of samples and standards was 100 μL. Injection was done automatically using an auto-sampler. The spectra were acquired at 280 nm. In addition, five different standard curves were prepared using different concentrations of commercial phenolic acids (1, 10, 20, 30, 40, 50 μg/mL) including p-coumaric acid, ferulic acid, gallic acid, sinapic acid, and cinnamic acid.

###  Antibacterial Activity of Plant Extracts

 The antibacterial activity of the date seed extracts against MRSA was evaluated using agar well diffusion. MRSA suspensions with turbidity equal to 0.5 McFarland were prepared in sterile normal saline from bacterial overnight liquid cultures in nutrient broth. Sterile cotton swabs were dipped in the bacterial suspensions and streaked over the entire surface of nutrient agar plates. In each prepared plate, wells (6 mm diameter) were made with the reverse side of the sterilized micropipette tips and then filled with 50 µL of the respective filter-sterilized extracts. The plates were incubated at 37 °C for 24 hours. The diameter of inhibition zones was measured in millimeters using a vernier caliper.All experiments were performed in triplicate.

###  Antioxidant Activity and Scanning Electron Microscopy 

 The antioxidant activity of date seed ethanolic extract was assayed using DPPH (2,2-Diphenyl-1-picrylhydrazyl) reagent. An aliquot of 0.1 mL of the different concentrations of the date seed extract (0.0625, 0.125, 0.250 and 0.500 mg/mL) was added to 3.9 mL of DPPH solution in a test tube. After incubation at 37 °C for 30 minutes, the absorbance of each solution was measured by a spectrophotometer (PerkinElmer, Germany) at 517 nm. The ability to scavenge DPPH radical was calculated by the following equation:

*DPPH radical scavenging activity (%) = [1-Absorbance of sample/ Absorbance of Standard] × 100*

 The changes in the morphological structures of bacterial cells treated with date seed extract was assessed using scanning electron microscopy (SEM) analysis. For this aim, 10 μL of the bacterial suspension was transferred on a cover glass and fixed by heat treatment. Then, chemical fixation was done using 100 μL of 2.5% glutaraldehyde. In the next step, ascending grades of ethanol (25%, 50%, 75%, 100%) were used for dehydration. Finally, samples were placed on the slide containing carbon glue coated with a thin layer of gold and placed in Zeiss EVO18 SEM (Germany) for imaging.

###  Statistical Analysis

**Figure 1:**
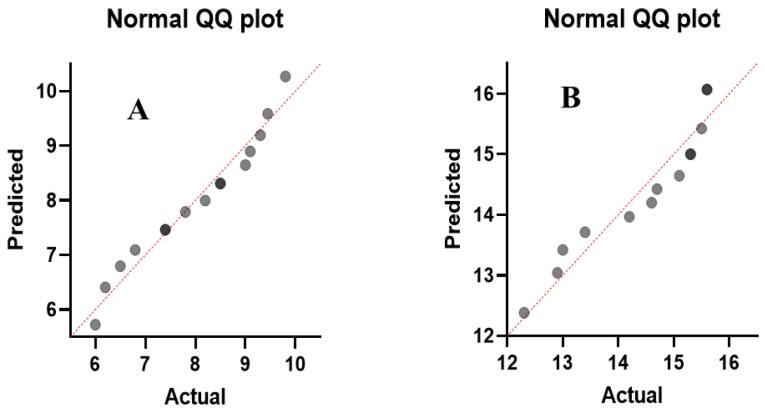
Normal Q-Q Plot of Data. (A) Growth inhibition zone values of Zahidi date; (B) Growth inhibition zone values of Khastawi date

 Data were analyzed by the SPSS (Statistical Package for the Social Sciences) statistic software version 25 using one-way ANOVA followed by Tukey test. A *P* value < 0.05 was considered statistically significant. Normal distribution of data was analyzed using GraphPad Prism 8.0 and Q-Q plots are depicted in Figure 1.

## Results

###  HPLC Analysis of Phenolic Profile

####  Standard Solutions

**Figure 2:**
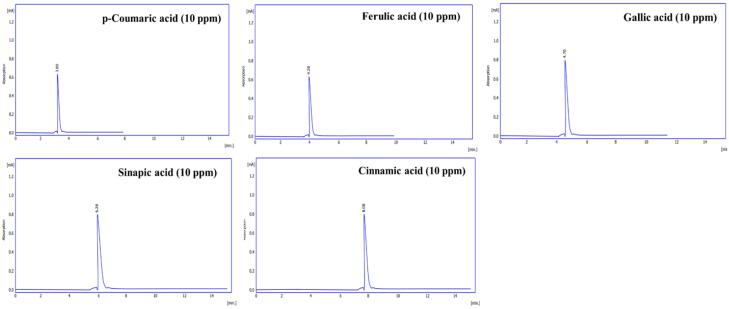
HPLC Chromatogram of Standard Phenolic Acids Solutions

 To determine the concentration of phenolic acids in Zahidi and Khastawi date seed extracts through HPLC, the chromatogram of standard solutions including ferulic acid, cinnamic acid, sinapic acid, gallic acid and p-coumaric acid were provided. Figure 2 demonstrates the HPLCchromatogramof standard solutions. Detailed data obtained from HPLC analysis of standard phenolic acids solutions including retention time, peak area and peak height are given in Table 1.

**Table 1:** Detailed Data Obtained from HPLC Analysis of Standard Phenolic Acids Solutions

**Phenolic acid **	**Retention Time (min)**	**Area (mAU.s)**	**Height (mAU)**	**Area (%)**	**Height (%)**	**W05 (min)**
p-Coumaric acid	3.80	1542.98	630.25	100	100	0.25
Ferulic acid	4.28	1895.08	633.25	100	100	0.25
Gallic acid	4.70	2577.49	794.15	100	100	0.25
Sinapic acid	6.28	2415.98	791.00	100	100	0.25
Cinnamic acid	8.08	1632.08	792.65	100	100	0.25

###  Date Seed Ethanolic Extract

**Figure 3:**
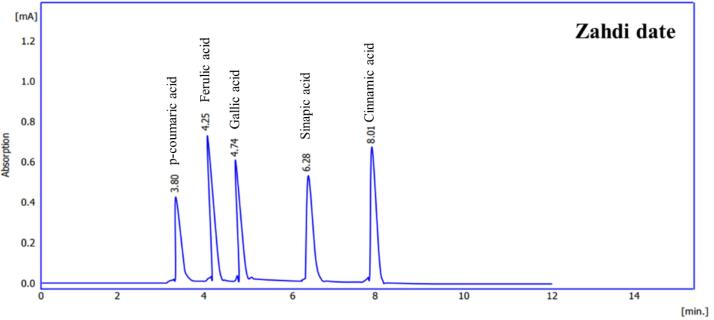
HPLC Chromatogram of Phenolic Acids of Zahidi Date Seed Extract

**Figure 4:**
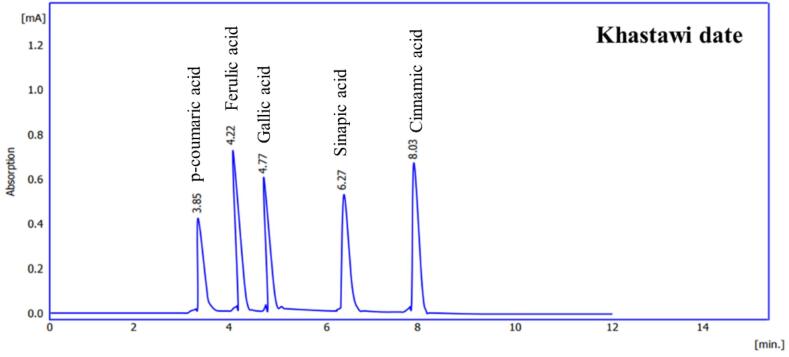
HPLC Chromatogram of Khastawi Date Seed Extract

 The phenolic profile of Zahidi and Khastawi date seed ethanolic extract was determined using HPLC analysis (Figures 3 and 4). The samples of the both date cultivars represented a similar chromatogram pattern for the quantified phenolic acids.

 Detailed data acquired in HPLC analysis of phenolic acids of Zahidi and Khastawi date seed extracts are given in Table 2.

**Table 2:** Detailed Data Obtained in HPLC Analysis of Phenolic Acids of Zahidi and Khastawi Date Seed Extracts.

**Phenolic acid**	**Retention Time (min)**	**Area (mAU.s)**	**Height (mAU)**	**Area (%)**	**Height (%)**	**W05 (min)**
**Zahidi Cultivar**						
p-Coumaric acid	3.80	24598.28	380.15	15.00	15.00	0.15
Ferulic acid	4.25	33652.44	780.65	25.00	25.00	0.25
Gallic acid	4.74	30665.08	620.33	20.00	20.00	0.20
Sinapic acid	6.28	27445.98	570.51	20.00	20.00	0.20
Cinnamic acid	8.01	30669.14	620.33	20.00	20.00	0.20
**Khastawi Cultivar**						
p-Coumaric acid	3.85	28652.08	382.46	15.00	15.00	0.15
Ferulic acid	4.22	39652.14	782.66	25.00	25.00	0.25
Gallic acid	4.77	32665.49	622.49	20.00	20.00	0.20
Sinapic acid	6.27	29652.32	571.65	20.00	20.00	0.20
Cinnamic acid	8.01	30669.14	620.33	20.00	20.00	0.20

 Determination of phenolic acid concentrations in the samples by HPLC showed that gallic acid was observed in the highest concentration in both Zahidi and Khastawi date seed extract at 25.89 and 30.25 µg mg^-1^, respectively. Also, cinnamic acid was identified in the lowest concentration in the samples of both cultivars (Table 3).

**Table 3:** Concentrations of Phenolic Acid in Zahidi and Khastawi Date Seed Extract Measured by HPLC

**Phenolic acid**	**Zahidi date (µg mg**^-1^)	**Khastawi date (µg mg**^-1^)
Gallic acid	33.65	41.56
p-Coumaric acid	25.89	30.25
Ferulic acid	16.99	20.14
Sinapic acid	14.56	18.99
Cinnamic acid	10.59	13.69

###  Evaluation the Anti-MRSA and Antioxidant Effects of Date Seed Extract

**Figure 5:**
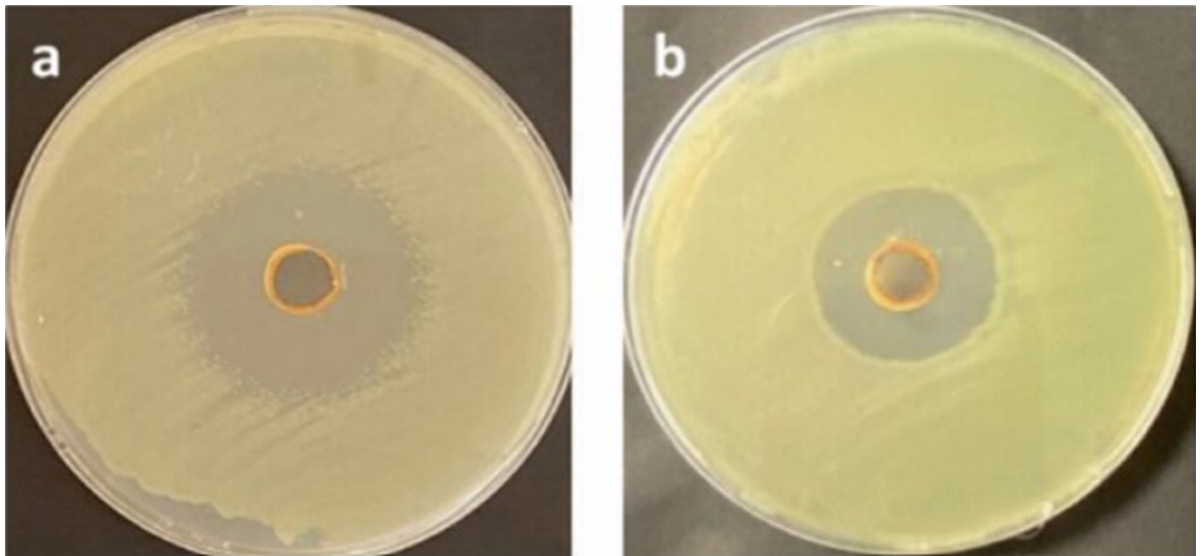
Zone of Growth Inhibition in MRSA Isolate Induced by Khastawi (a) and Zahidi (b) Date Seed Extracts

 The anti-MRSA activity of Zahidi and Khastawi date seed extracts was tested *in vitro* by agar well diffusion. Bacterial growth inhibition zones were measured at the end of the incubation period. The results of antibacterial activity assessment showed that the diameters of the inhibition zones of Khastawi and Zahidi seed extract were 14 ± 0.21 mm and 8 ± 0.13 mm, respectively. In other words, the antibacterial properties of Khastawi date were significantly (*P* value = 0.031) higher than Zahidi date (Figure 5).

**Figure 6:**
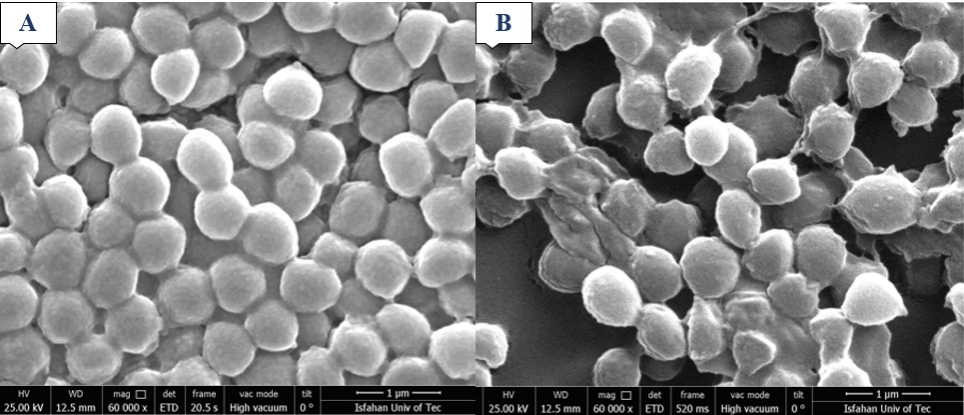
Scanning Electron Microscopy of MRSA Treated with the Khastawi Date Seed Extract. A) Untreated Sample and B) Treated Sample

 The analysis of antibacterial effects of Khastawi date seed extract using SEM revealed that in the control condition (Figure 6A), bacterial cells had normal shape but in the treated sample (Figure 6B), numerous cells were lysed and some of them had a hole on their cell wall.

**Figure 7:**
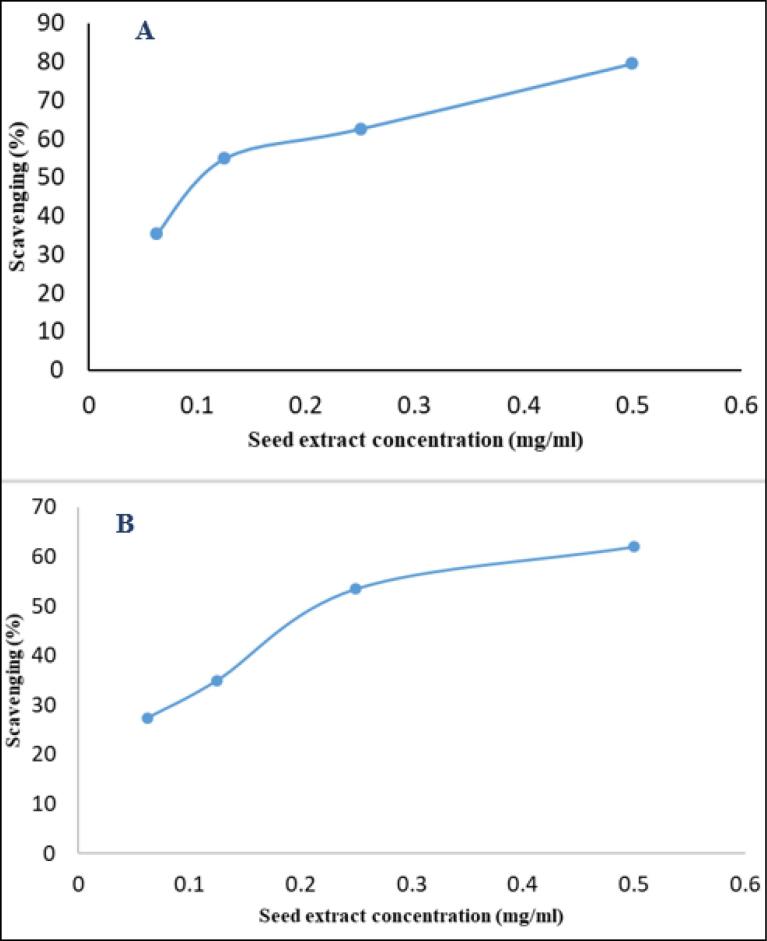
Antioxidant Activity of Khastawi (A) and Zahidi (B) Date Seed Extracts

 As depicted in Figure 7, the antioxidant activity of both Khastawi and Zahidi date seed extracts increased in a concentration-dependent manner. Additionally, the antioxidant capacity of the Khastawi date seed extract was higher than Zahidi. The maximum DPPH scavenging percentage was 79% and 62% for the Khastawi and Zahidi date seed extracts, respectively.

## Discussion

 Date seeds are usually considered as the waste of date fruit production process and are disposed or may be used as animal feed in some cases. The seeds comprise 10%–15% of date fresh fruit weight, and are known as a rich source of total polyphenols and phenolic acids including caffeic acid, chlorogenic acid, p-coumaric acid, ferulic acid, gallic acid, syringic acid, and vanillic acid. It is worth noting that the phenolic content of plants is affected by the environmental conditions. For example, Brahmi et al suggested that plants grown on nutrient-poor soils accumulate higher phenolic contents than those grown on fertile soils.^22,23^ Beneficial effects of date seeds in terms of health-promoting activities including antioxidant, anti-inflammation, antidiabetic, antibacterial, and antiviral functions have been explored in several reports.^24^ The main objective of the present study was to investigate the relationship between phenolic contents in date seed extracts and their antibacterial activity. According to our results, it seems that there is a relationship between gallic acid titer and antibacterial activity of date seed extracts because the gallic acid content in Khastawi date seed extract was higher than that of Zahidi date seed extracts and its antibacterial activity was also higher (Table 4). Previous studies have confirmed the role of polyphenol compounds in the beneficial properties of date seeds. Flavan-3-ols have been reported as the major group of polyphenols in date seeds present as polymeric proanthocyanidins. The polyphenol content of date seed is estimated about 51.1g/kg which is higher than other polyphenol-rich sources such as tea, grapes and flaxseeds.^25^

**Table 4:** Relationship between Gallic Acid Concentration and Bioactivities of Date Seed Extracts

	**Zahidi Date** **(Mean±SD)**	**Khastawi Date** **(Mean±SD)**	***P *** **Value **	**95% CI for the Difference Between Two Groups **
Concentrations of gallic acid (µg mg^-1^)	33.65	41.56	—	—
Growth inhibition zone (mm)	8 ± 0.13	14 ± 0.21	0.031	(5.46, 6.54)
DPPH scavenging (%)	62 ± 1.23	79 ± 2.3	0.004	(11.37, 22.63)

 In the study by Baghbani and Shirazinejad, the antimicrobial property of different concentrations of date seed aqueous extract (1, 0.05, 0.05 and 0.2 mL/µg) against *S. typhimurium* was evaluated. Minimum inhibitory concentration (MIC) and minimum bactericidal concentration (MBC) were 0.08 and 0.4 mg/mL, respectively. HPLC fractionation of the extract detected rutin as the major compound of the extract, followed by catechin and sinapic acid. Chlorogenic acid, vanillin, p-coumaric acid and gallic acid were in the next ranks.^26^ The findings of Selim et al indicated that the methanolic extract of date pits exhibited high levels of total phenols (17.38 mg gallic acid per g) and flavonoids (5.324 mg quercetin equivalent per g). The HPLC analysis of the date pit extract revealed the presence of six phenolic compounds, with significant amounts of gallic acid. The purified gallic acid at a concentration of 200 µg/mL completely inhibited the growth of *S. aureus*.^27^ Also, in the current study, gallic acid was found as the predominant phenolic acid in the seed extract of Zahidi and Khastawi date cultivars. This finding was also in accordance with the result of the research by Al Harthi et al, who reported gallic acid as the major phenolic acid in all four studied date varieties.^28^ Alrajhi et al evaluated the antibacterial activity of date seed cake extract provided by cold extraction method using hexane and ethyl-acetate against gram-negative and gram-positive bacteria. MRSA strain (with an inhibition zone of 20 mm diameter) and *Proteus mirabilis* showed the highest sensitivity against date seed extracts among the tested gram-positive and gram-negative bacteria, respectively.^29^ In another study, methanolic extracts of Ajwa date seed and fruit were provided and their different potential activities including antioxidant, anti-hemolytic, anti-proteolytic, and antibacterial activities were assessed. Significant inhibition zone against *S. aureus*, *E. coli*, *K. pneumonia*, *P. aeruginosa*, and *E. faecalis* was seen for Ajwa date seed extract at the concentration of 100 mg/mL with diameters of 19 mm, 15 mm, 16 mm, 14 mm, and 16 mm, respectively.^30^ The antibacterial properties of Zahidi date seed extract associated with Halawi and Khadrawiand date seed extracts were assessed against eighth pathogenic bacterial strains by Aljazy and colleagues. Comparing the antimicrobial activity of aqueous, ethanolic and methanolic extracts of Zahidi date seed extract revealed that ethanolic and methanolic extracts showed stronger activity than the aqueous extract. The largest inhibition zone of methanolic and ethanolic extracts were observed against *Pseudomonas aeruginosa* (16 mm diameter) and *Proteus mirabilis* (22 mm), respectively. The inhibition zone diameter for *S. aureus* was 8 mm. ^31^ In the study by Muhialdin and colleague, the antimicrobial activity of Khastawi date fruits fermented with *Lactobacillus plantarum* was evaluated. The results indicated broad-spectrum antimicrobial activity of fermented date fruit against *Aspergillus niger* (90.85%), *Aspergillus flavus* (92.86%), *E. coli* (13 mm) and *S. aureus* (15 mm).^32^ Our results showed that both date seed extracts had significant antioxidant activity. However, the antioxidant activity of the Khastawi date seed extract was higher than Zahidi date seed extract, which can be attributed to its higher content of phenolic acids. A similar observation was made about the antibacterial activity of these extracts. Furthermore, SEM analysis suggested that date seed extract caused a significant alteration in the morphology of the treated bacterial cells. Generally, the main mechanism of the antibacterial activity of plant extracts has not been discovered. However, it has been hypothesized that disruption of the cell membrane is the major mechanism.^33^ The results of the present study provide valuable insights into the antibacterial and antioxidant activity of date seed extracts. However, it should be noted that in this study, only two types of dates were used and their biological effects were investigated against only one specific bacterial strain. Another limitation of this study was impurities present in date seed extracts. This issue can be important because the presence of other unknown compounds in plant extracts, in addition to phenolic compounds, can affect their biological properties.

## Conclusion

 While date seeds appear to be a troublesome waste material, they are identified as a valuable source of health beneficial phytochemicals and bioactive compounds. In this study, the ethanolic extract of two date cultivars, Zahidi and Khastawi, were explored for some key phenolic acids quantification by HPLC. Gallic acid and cinnamic acid were respectively found to be in the highest and lowest concentration in both cultivars. Also, the growth inhibition effect of Khastawi date was higher than Zahidi date seed against MRSA. In conclusion, date seed extract is a significant source of nutraceuticals which can be used for further investigations to develop novel antibiotic substances. However, further studies are necessary to find out the exact antibacterial mechanism of these compounds; for example, the molecular docking method to evaluate the affinity of phenolic acid compounds to specific target proteins on pathogenic bacteria and real-time PCR technique to investigate the alterations in the expression of virulence genes in pathogens treated with date seed extracts could be helpful for this purpose. Additionally, only one type of bacterial strain was used to evaluate the antibacterial activity of date seed extracts while determination of the size of the inhibition zones or the MIC values across multiple strains of bacteria is necessary to determine that whether the plant extract has broad-spectrum activity or narrow-spectrum activity. Finally, the safety or toxicity against human cells need to be evaluated, as well.
